# Supplementary material for: Overcoming the water oxidative limit for ultra-high-workfunction hole-doped polymers
Source: Nat Commun. 2021 Jun 7;12:3345. doi: 10.1038/s41467-021-23347-x (PMC8184950; doi:10.1038/s41467-021-23347-x)
Supplement: Supplementary file 1 — Supplementary Information [file 41467_2021_23347_MOESM1_ESM.pdf]

Overcoming the water oxidative limit for ultra-high-workfunction hole-doped polymers

Qi-Mian KOH,[1‡] Cindy Guanyu TANG,[2‡] Mervin Chun-Yi ANG,[1] Kim-Kian CHOO,[1] Qiu-Jing SEAH,[1] Rui-Qi PNG,[2*] Lay-Lay CHUA,[1,2*] Peter K.H. HO[2]

[1] Department of Chemistry, National University of Singapore, Lower Kent Ridge Road, S117552, Singapore

[2] Department of Physics, National University of Singapore, Lower Kent Ridge Road, S117550, Singapore

‡ These authors contributed equally to the work.

* Correspondence to:
ruiqi@nus.edu.sg (R.Q.P.) or chmcll@nus.edu.sg (L.L.C.)

Supplementary Information

Contents

1. Supplementary Figures 2

2. Supplementary Tables 20

3. Supplementary Notes 30

4. Supplementary References 33

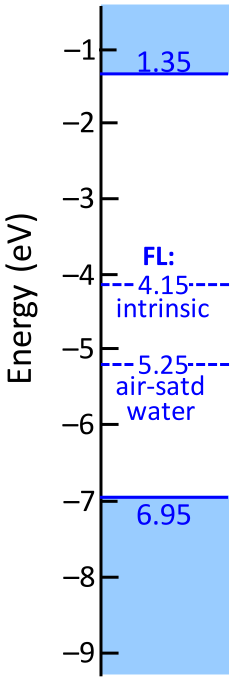

Supplementary Figure 1. Energy band diagram of liquid water, constructed from literature. The electrode potential of air-saturated, pH-neutral liquid water is $\mathcal{E}$ = 0.815 V *vs* standard hydrogen electrode. This corresponds to 5.25 eV on the vacuum scale. The intrinsic Fermi level (FL) of water is at 4.15 eV, based on its Gibbs free energies for adiabatic ionization ($\Delta G_{a,\ell}^{ion}$ = 6.95 eV) and adiabatic electron affinity ($\Delta G_{a,\ell}^{EA}$ = 1.35 eV).[1] The corresponding vertical ionization energy and electron affinity are 10.2 and 0.0 eV, respectively.[2,3]

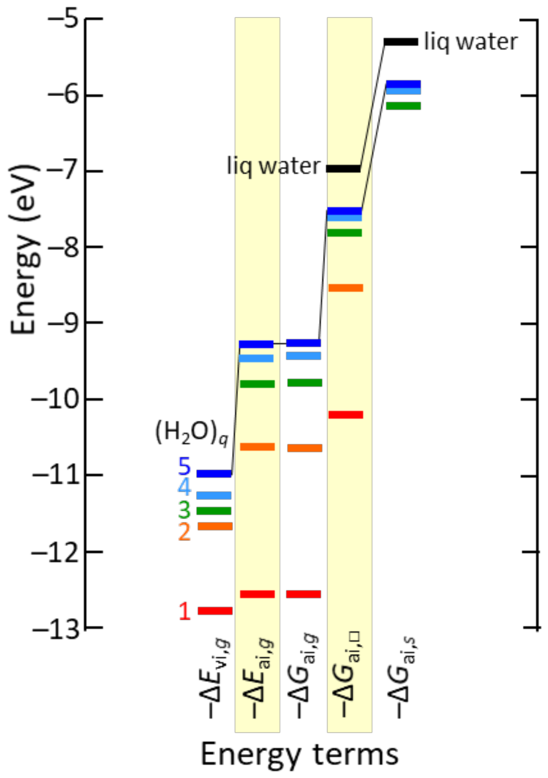

Supplementary Figure 2. Donor level and Fermi level of water clusters $(H_2O)_q$. Computed values are for the ionization process: $(H_2O)_{q\ (g)} \rightarrow H^+(H_2O)_{q-1}\ HO\bullet_{\ (g)} + e^-_{\ (g)}$, plotted relative to vacuum level. $\Delta E_{vi,g}$ is the change in gas-phase internal energy for vertical ionization at 0 K. $\Delta E_{ai,g}$ is the corresponding quantity for adiabatic ionization at 0 K. $\Delta G_{ai,g}$ is the change in Gibbs free energy for adiabatic ionization at 298 K. $\Delta G_{ai,\square}$ is the corresponding quantity in the hole-doped organic semiconductor matrix, by correcting for polarization and carrier screening. The cavity size is given by the size of the water cluster. Matrix parameters: dielectric constant, 3.0; carrier screening length, 12 Å. $-\Delta G_{ai,s}$ gives the Fermi level of the redox species in their specified states. Bulk limit: The adiabatic ionization energy of liquid water is 6.95 eV: $H_2O_{\ (\ell)} \rightarrow H^+_{\ (aq)} + HO\bullet_{\ (aq)} + e^-_{\ (g)}$. The follow-on reaction has a free-energy change of –1.665 eV: $HO\bullet_{\ (aq)} \rightarrow \frac{1}{4}\ O_{2\ (g)} + \frac{1}{2}\ H_2O_{\ (aq)}$. These give the electrode potential of liquid water under 1-bar O_2 to be –5.28 eV on the vacuum scale, which corresponds to $\mathcal{E}$ = 0.845 V *vs* standard hydrogen electrode. Assuming same reaction for water clusters, and same free-energy change, the Fermi level for $(H_2O)_5$ lies at –5.88 eV.

TMA$^+$ PF_6^- $(H_2O)_p$

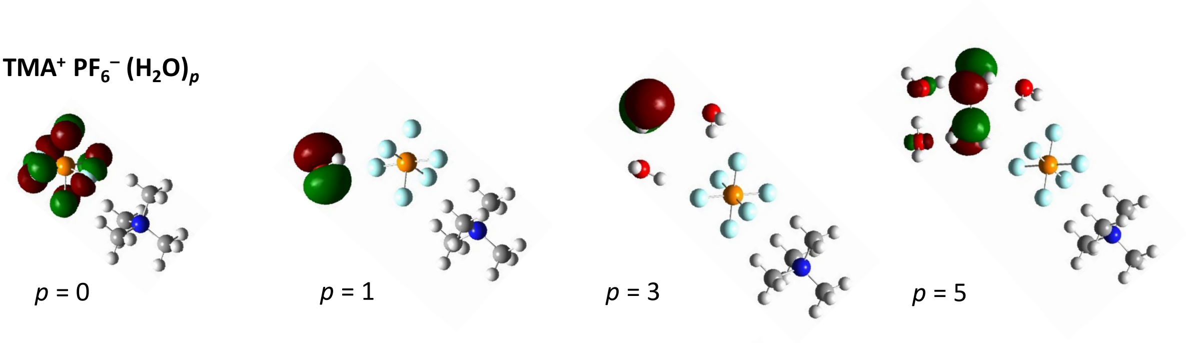

TMA$^+$ $(CF_3SO_2)_2N^-$ $(H_2O)_p$

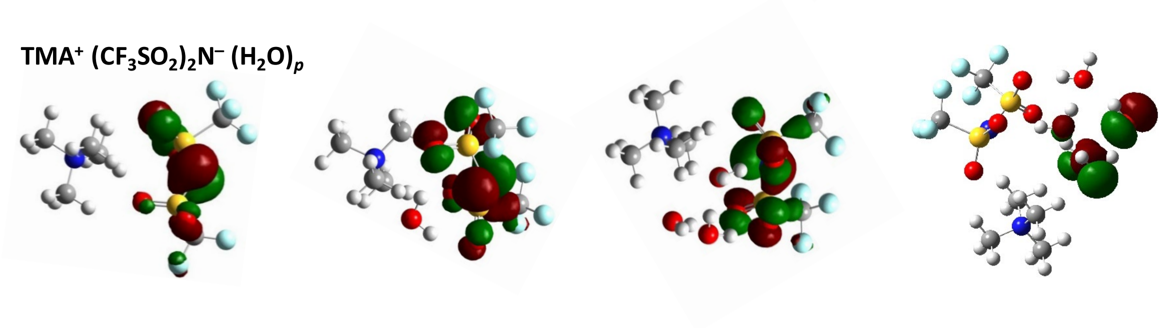

TMA$^+$ $(CH_3SO_2)(CF_3SO_2)N^-$ $(H_2O)_p$

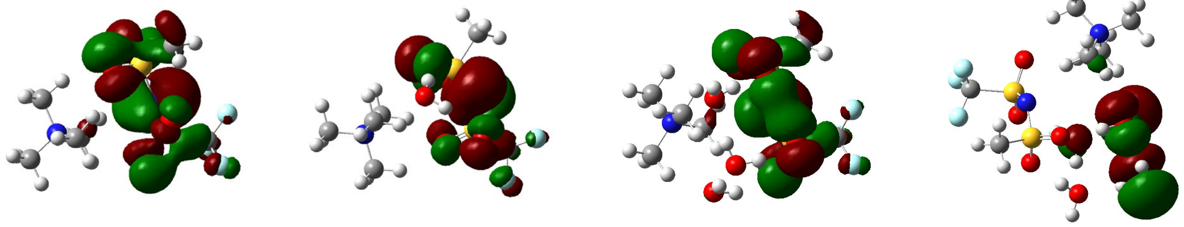

TMA$^+$ $CF_3SO_3^-$ $(H_2O)_p$

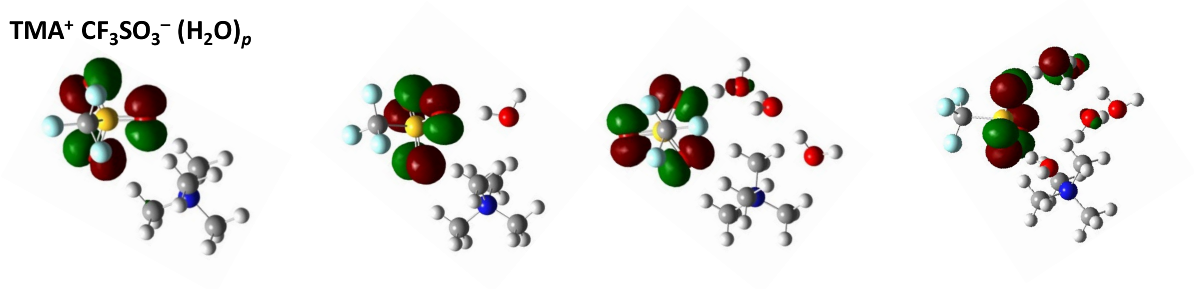

TMA$^+$ $CH_3SO_3^-$ $(H_2O)_p$

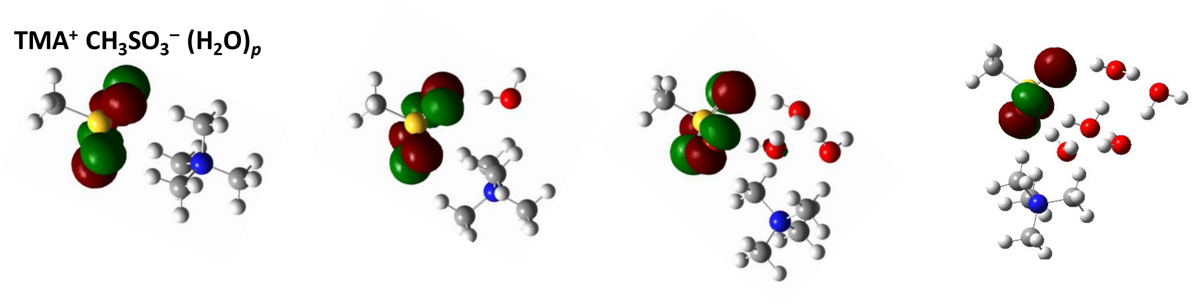

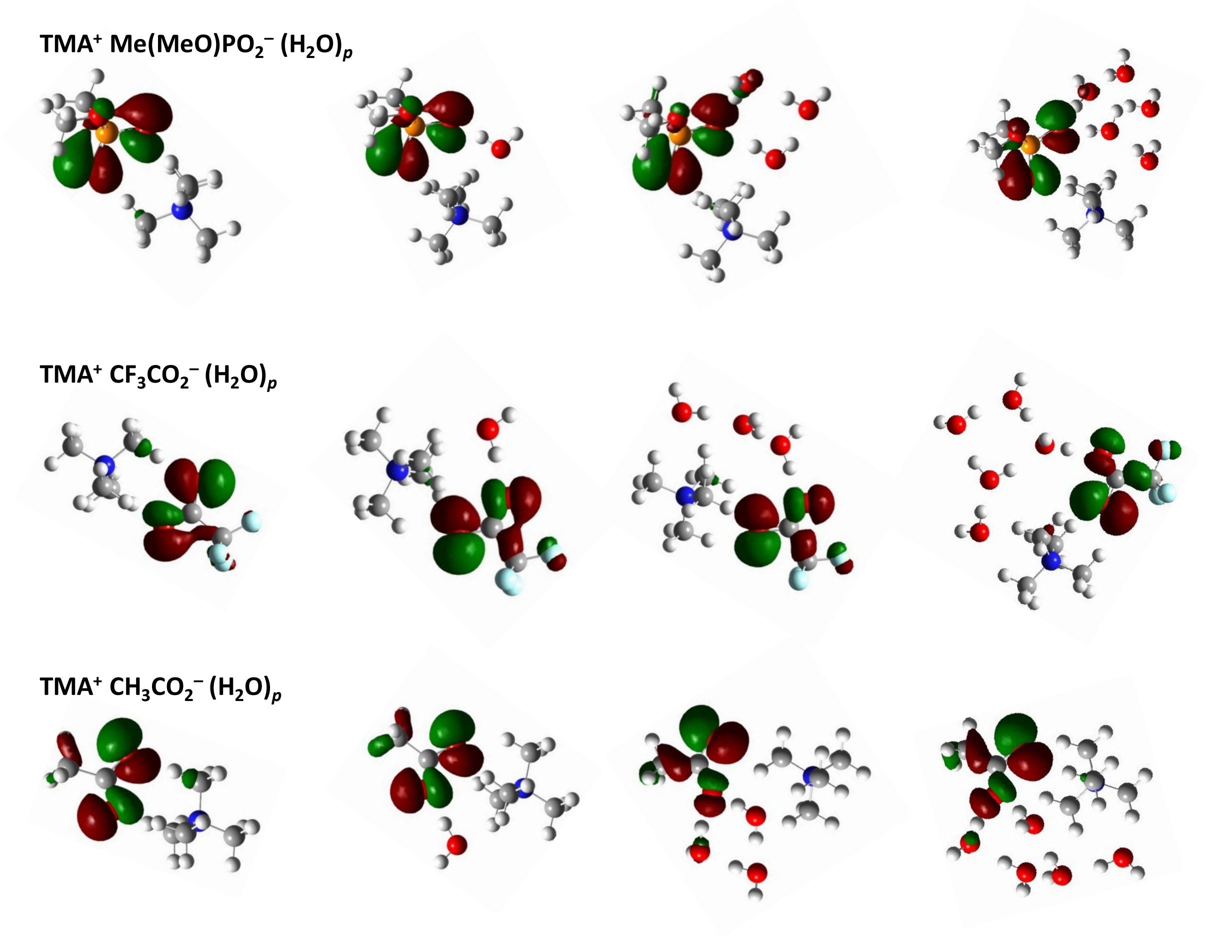

Supplementary Figure 3. Computed HOMO wavefunction and geometry in the ground state. HOMO is the highest-occupied-molecular-orbital. It reveals where the highest-energy electron resides in the ground state. Computations were performed at DFT/CAM-B3LYP/6-31++G(*d*,*p*). TMA$^+$ is tetramethylammonium ion, used as proxy for the positively-charged polymer backbone fragment. Atom legend: cyan, fluorine; grey, carbon; white, hydrogen; blue, nitrogen; red, oxygen; orange, phosphorus; yellow, sulfur.

TMA$^+$ PF_6^- $(H_2O)_p$

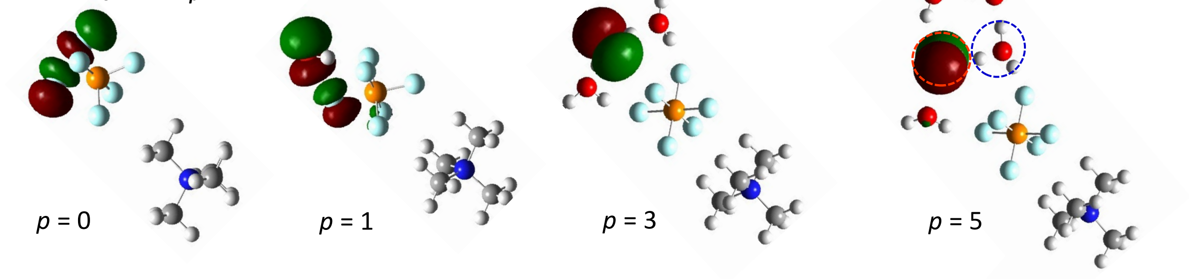

TMA$^+$ $(CF_3SO_2)_2N^-$ $(H_2O)_p$

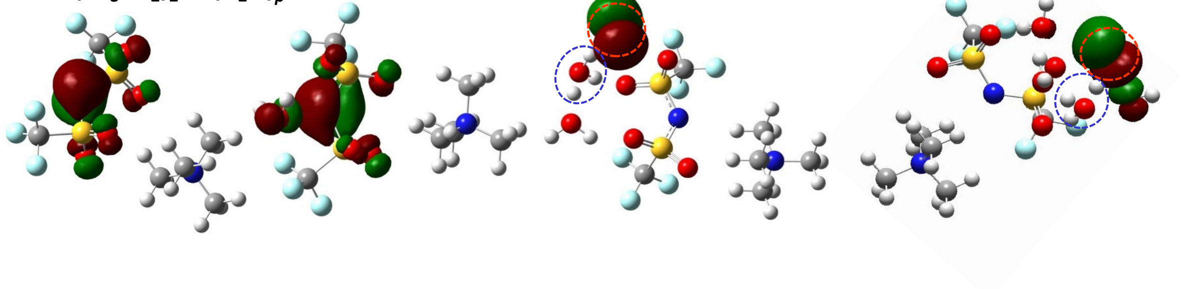

TMA$^+$ $(CH_3SO_2)(CF_3SO_2)N^-$ $(H_2O)_p$

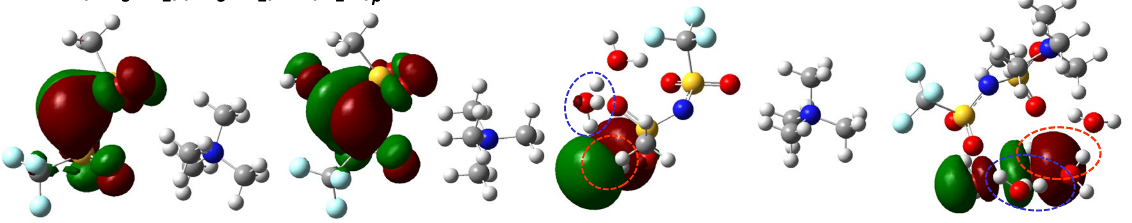

TMA$^+$ $CF_3SO_3^-$ $(H_2O)_p$

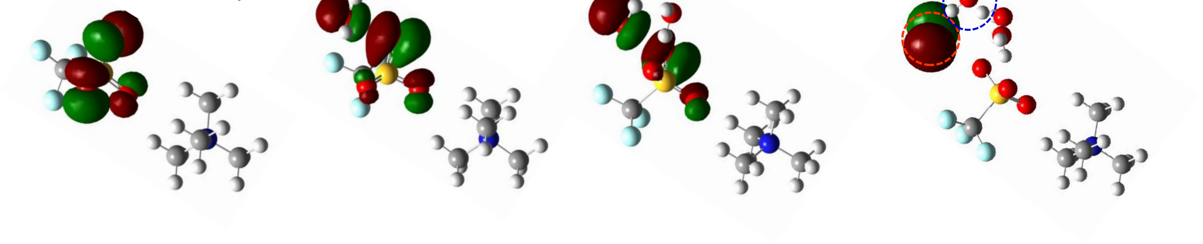

TMA$^+$ $CH_3SO_3^-$ $(H_2O)_p$

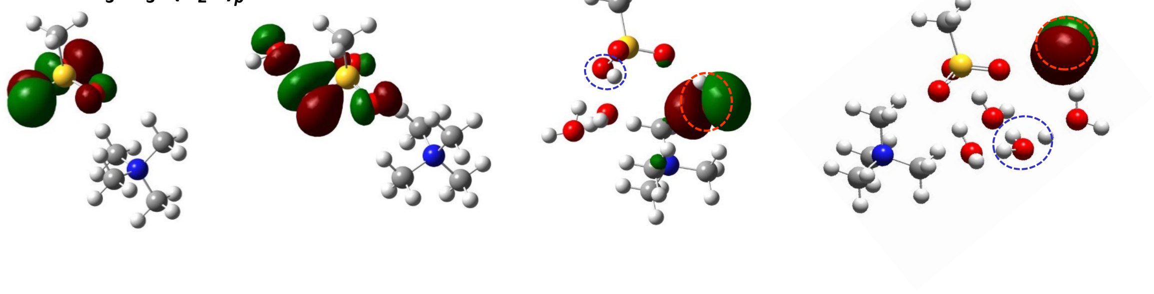

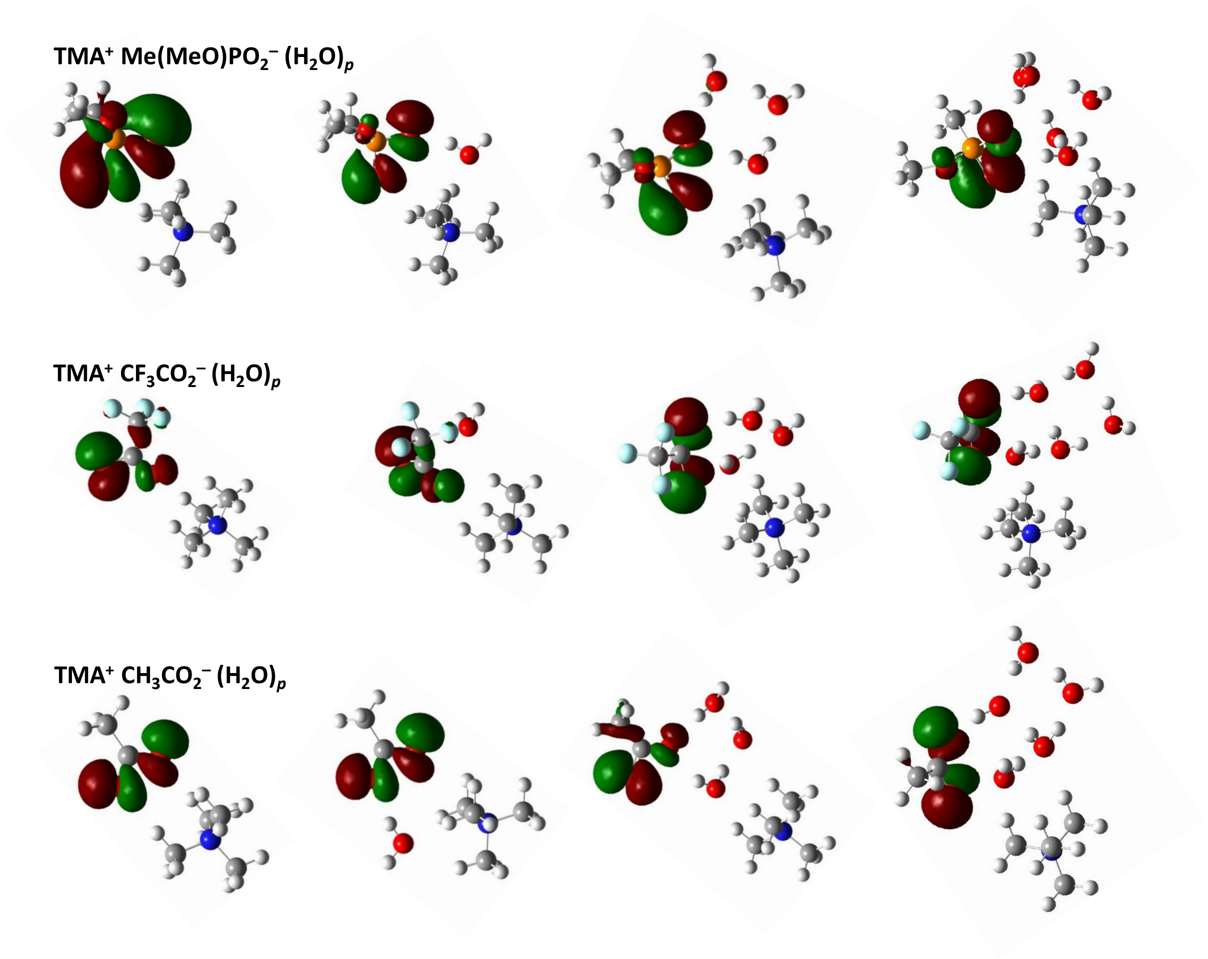

Supplementary Figure 4. Computed SOMO* wavefunction and geometry after one-electron detachment. SOMO* is the empty counterpart of the singly-occupied-molecular-orbital after electron detachment and full geometry relaxation. It reveals where the relaxed hole resides. Computations were performed at DFT/CAM-B3LYP/6-31++G(*d*,*p*). TMA^+ is tetramethylammonium ion, used as proxy for the positively-charged polymer backbone fragment. Dashed blue circle identifies hydronium or protonated anion; dashed orange circle identifies hydroxyl radical. Atom legend is the same as Supplementary Figure 3. Examination of wavefunctions reveals the following. For $p \in \{0, 1\}$, electron detachment occurs from the anion. For higher hydrated complexes, the product depends on the anion. For $CH_3SO_3^-$, $p = 3$, product is CH_3SO_3H; for $p = 5$, $CH_3SO_3^-\ H^+(H_2O)_4$. For $CF_3SO_3^-$, $p = 3$, product is anion radical; for $p = 5$, $CF_3SO_3^-\ H^+(H_2O)_4$. For PF_6^-, $(CF_3SO_2)_2N^-$ and $(CH_3SO_2)(CF_3SO_2)N^-$, $p = 3$, product is the anion…hydronium pair. For carboxylates, the product is anion radical.

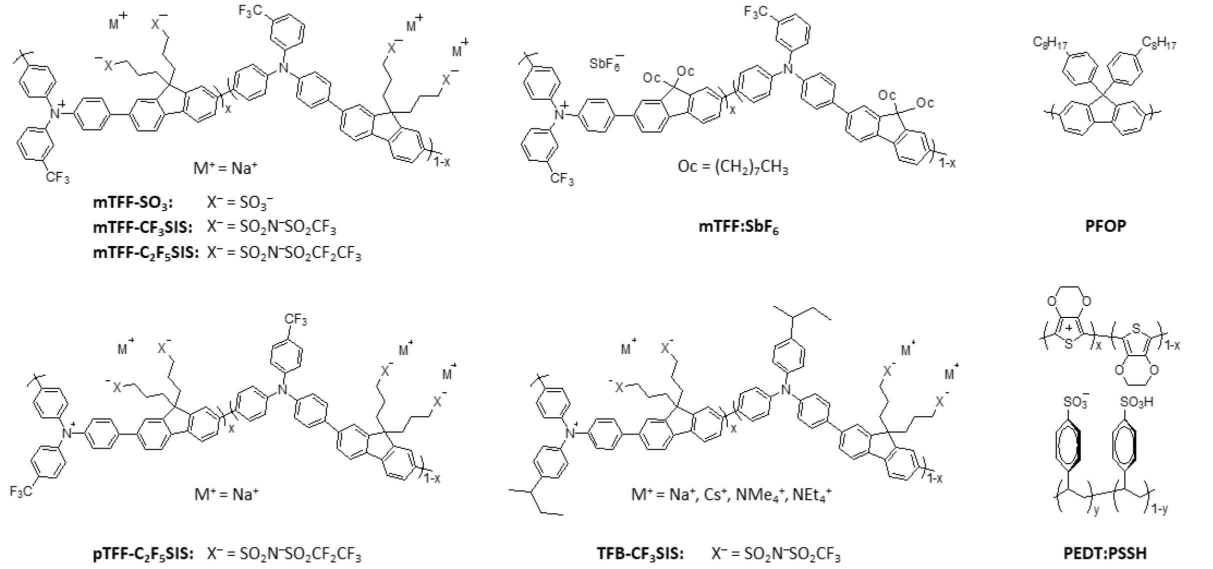

Supplementary Figure 5. Chemical structure of materials. The value of *x* denotes the doping level, in hole per repeat unit: $x = 0$, undoped; $x = 1$, fully-doped polymer.

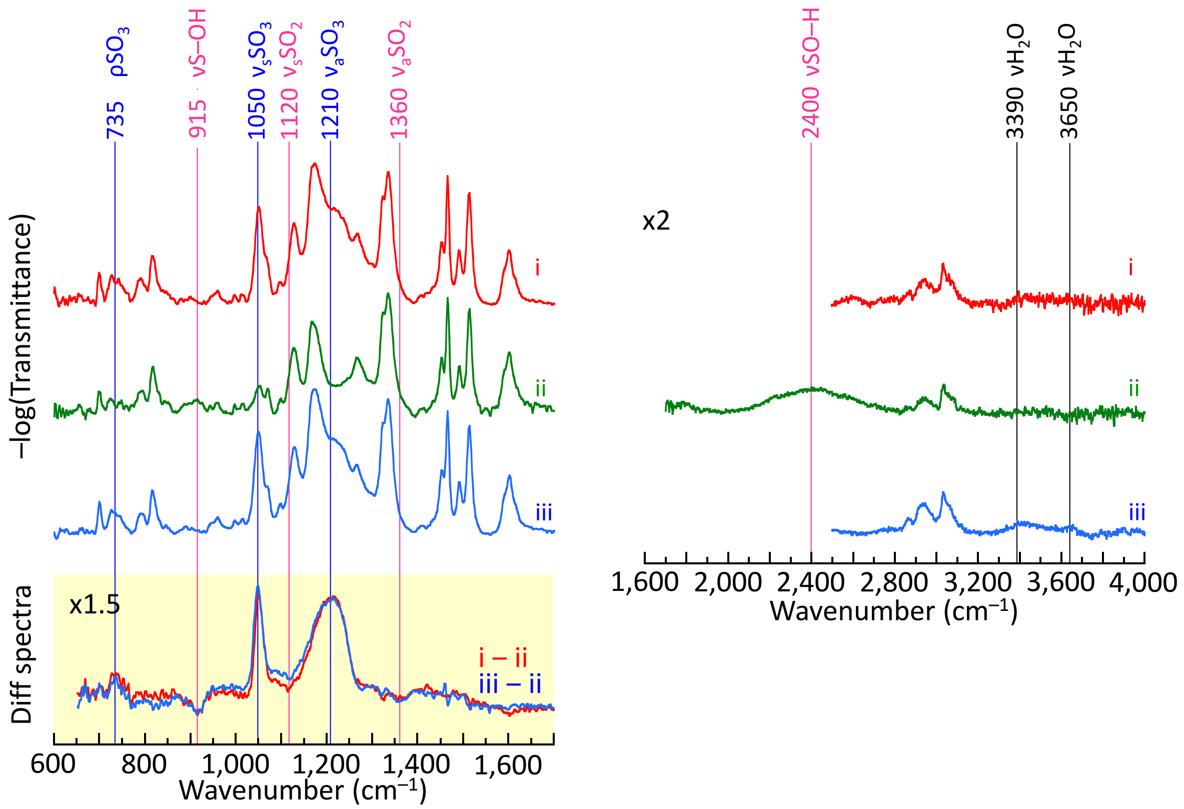

Supplementary Figure 6. Transmission FTIR spectra of undoped mTFF-SO_3 films. (White panel) Spectra of: (i) Na^+ salt form, (ii) covalent acid (protonated anion) form, and (iii) after doping/thermal-dedoping cycle and Na^+ exchange. **(Yellow panel)** Difference spectra. All films were annealed on hotplate to 220°C for 15 min in the glovebox before spectra collection. Blue lines mark the vibrational modes of the sulfonate anion; magenta lines, sulfonic acid group; black lines, sorbed molecular water. **Methodology:** mTFF-SO_3 was synthesized and purified to give the Na^+ salt form. This was spin-cast to give the Na^+ salt film (i), which was then contacted with 1-mM aqueous trifluoromethanesulfonic acid for 30 s, and spin-dried to produce the acid film (ii). Another Na^+ salt film was contacted with 1-mM $NOSbF_6$ in anhydrous acetonitrile for 30 s, washed with anhydrous acetonitrile, and spin-dried to produce the self-compensated, hole-doped film. This film was heated on a hotplate to 180°C for 15 min in the nitrogen glovebox to causing complete de-doping, then contacted with a 1-mM aqueous sodium hydroxide for 10 s to exchange protons to Na^+, and spin-dried to give film (iii). Its FTIR spectrum is indeed identical to (i). This confirms the absence of irreversible chemical changes during the doping/thermal-dedoping cycle. At the same time, it produces reference spectral positions for the sulfonate anion and sulfonic acid group.

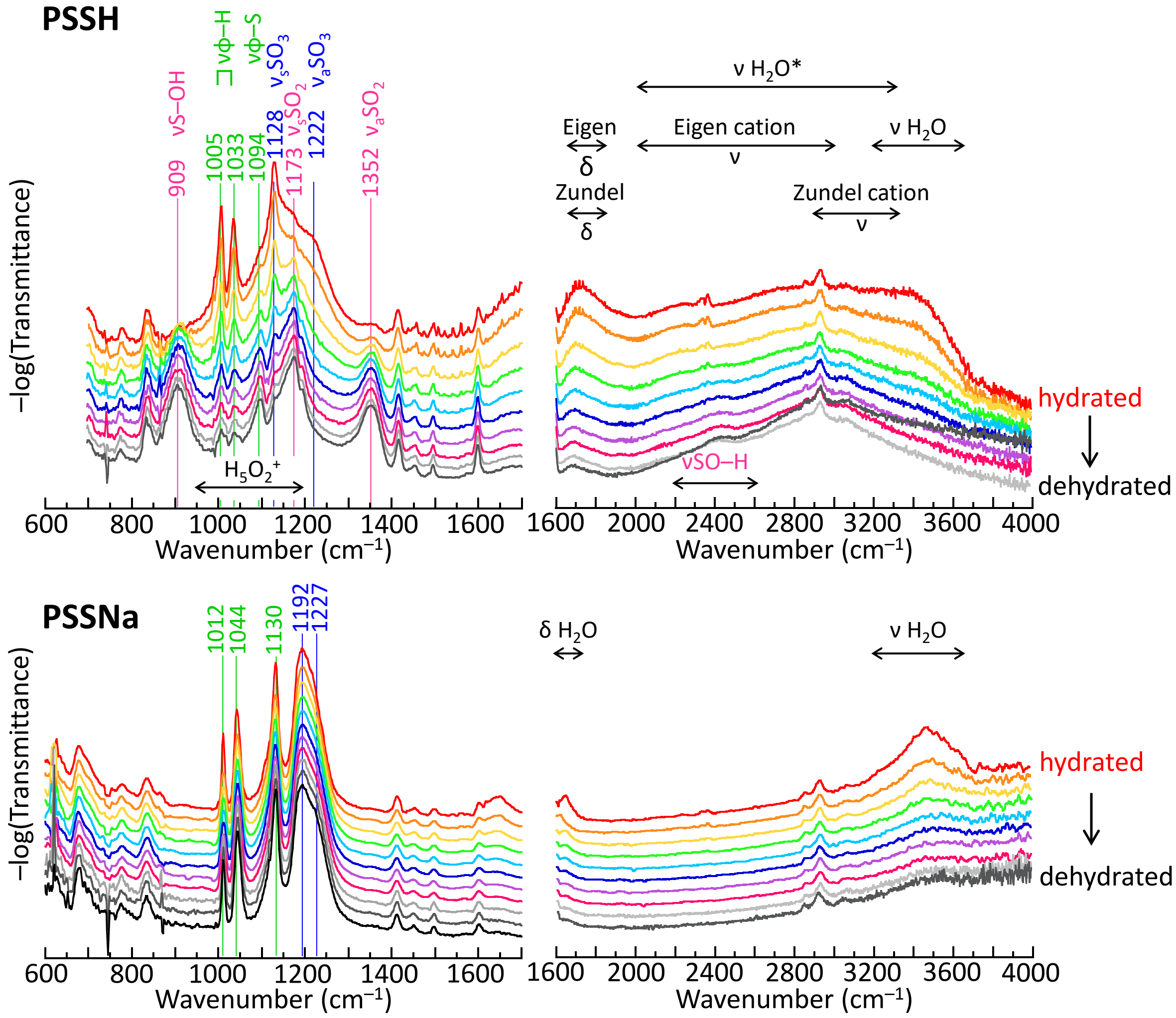

Supplementary Figure 7. Transmission FTIR spectra of poly(styrenesulfonate) films. (Top) Poly(styrenesulfonic acid) (PSSH), **(bottom)** poly(styrenesulfonate sodium) (PSSNa). **Methodology:** The films were cast in ambient and transferred to the nitrogen-purged sample chamber of the FTIR spectrometer. Their spectra were recorded as a function of time in flowing nitrogen as the degree of hydration decreased. The sulfonic acid group in PSSH comprises $–SO_3^-$ $H^+(H_2O)_p$ initially, which transforms into a network of strongly hydrogen-bonded $–SO_3H...OH_2$ and finally $–SO_3H$ as it dehydrates. In contrast, the sulfonate anion in PSSNa remains unchanged as it dehydrates.

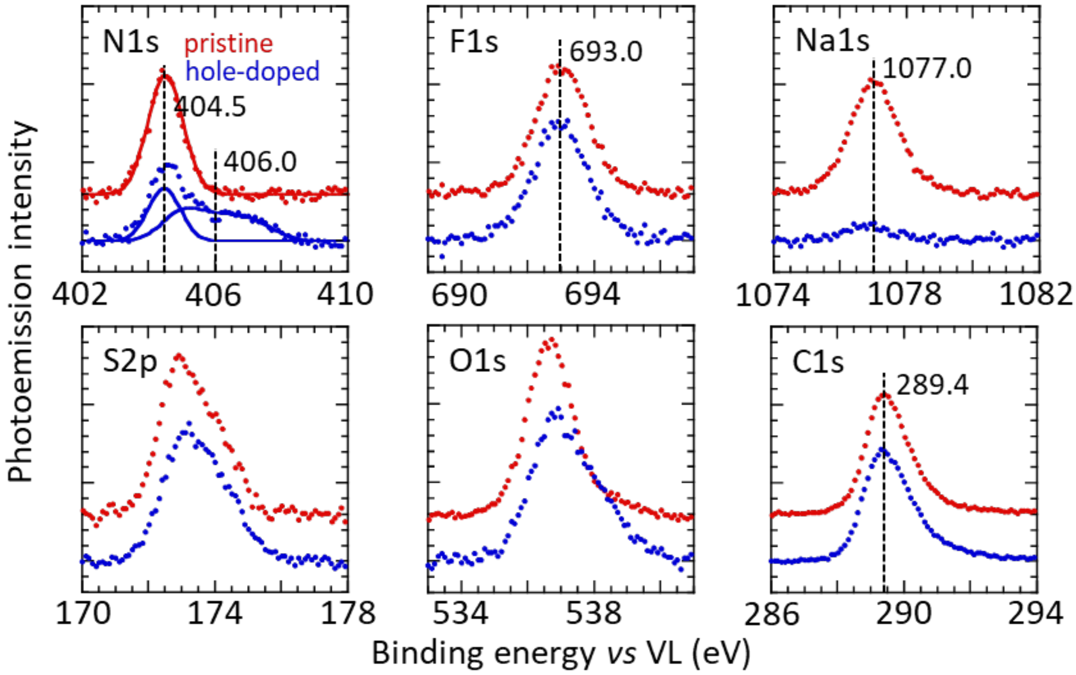

Supplementary Figure 8. X-ray photoemission spectroscopy of mTFF-SO_3 films. All binding energies are referenced to vacuum level (VL). **Film preparation:** A 20-nm-thick mTFF-SO_3 film was spin-cast on Au-coated Si wafer, and analyzed as pristine film. Another film was hole-doped by contact with $NOSbF_6$ in anhydrous acetonitrile, washed with anhydrous acetonitrile, and spin-dried, all in the nitrogen glovebox, and analyzed as hole-doped film. **Analysis:** Following hole doping, (i) N1s core level shows loss of the neutral amine intensity (404.5 eV), and gain of positively-charged aminium intensity (centred 406.0 eV; multi-environment). (ii) Na1s intensity decreases by 90% instead of 50%, the excess loss due to proton substitution. (iii) S2p and O1s spectra show a higher binding-energy shoulder due to conversion of ionized $–SO_3^-$ Na^+ to un-ionized $–SO_2OH$ form.[4] (iv) No low-binding-energy F1s component is found, confirming SbF_6^- is not present in film.

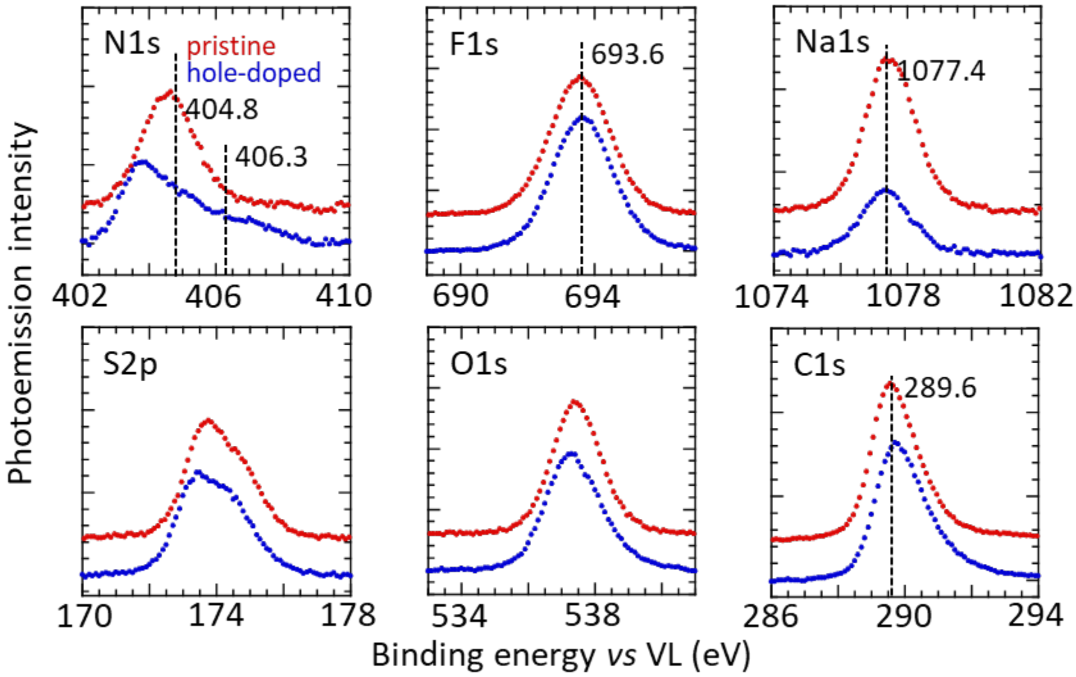

Supplementary Figure 9. X-ray photoemission spectroscopy of pTFF-C_2F_5SIS films. All binding energies are referenced to vacuum level. **Film preparation:** Undoped pTFF-C_2F_5SIS solution was spin-cast to give a 20-nm-thick film on Au-coated Si wafer, which was analyzed as pristine film. The solution was then hole-doped with $NOSbF_6$ (1.0 equiv) in anhydrous acetonitrile, purified by precipitation, and re-dissolved in anhydrous acetonitrile. This solution was spin-cast to give a 20-nm-thick hole-doped film. **Analysis:** Following hole doping, (i) N1s core level shows loss of the neutral amine intensity (404.8 eV), and gain of positively-charged aminium intensity (406.3 eV; multi-environment). Imide nitrogen (403.7 eV) remains. (ii) Na1s intensity decreases by 50%, close to theoretical value. (iii) S2p and O1s spectra are substantially unchanged. Analysis gives 1–1.5 H_2O per anion. (iv) No low-binding-energy F1s component is found, confirming SbF_6^- is not present in film.

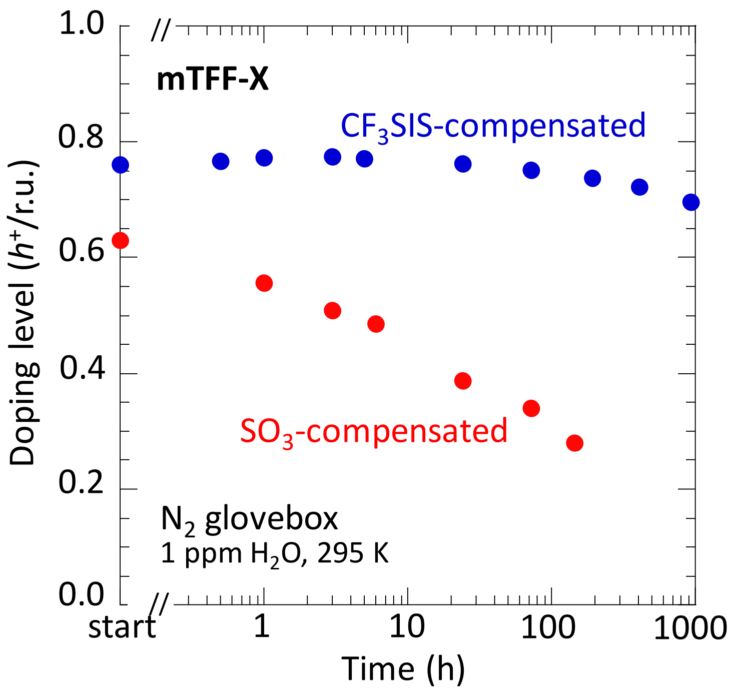

Supplementary Figure 10. Doping stability characteristics of hole-doped mTFF-X films in nitrogen glovebox. Doping-level stability with log(time) in nitrogen glovebox. Film thickness, 50 nm. Doping level was evaluated from neutral π→π* and polaron intensities, with estimated uncertainty of ± 0.05 h^+/r.u.

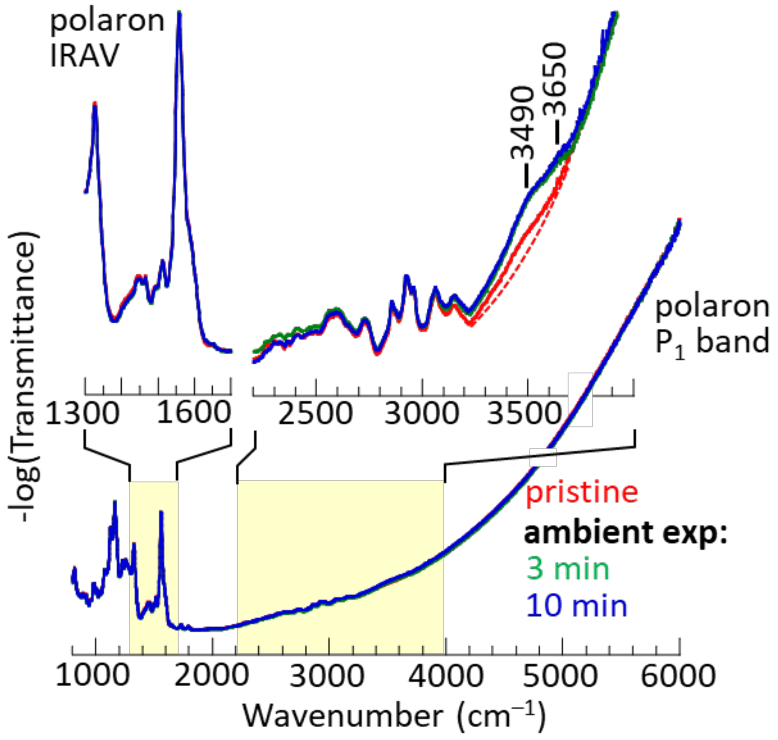

Supplementary Figure 11. Moisture sorption characteristics of hole-doped mTFF-CF_3SIS film. *In situ* FTIR spectra collected sequentially in vacuum (pristine) and after admitting ambient air (RH 65%, 295 K) into the sample chamber for 3 min and 10 min. Fast uptake of moisture occurs without triggering de-doping, in contrast to sulfonate film (Figure 3a, main article). The 3,490 and 3,650-cm^{-1} bands arise from molecular H_2O sorbed by the film.

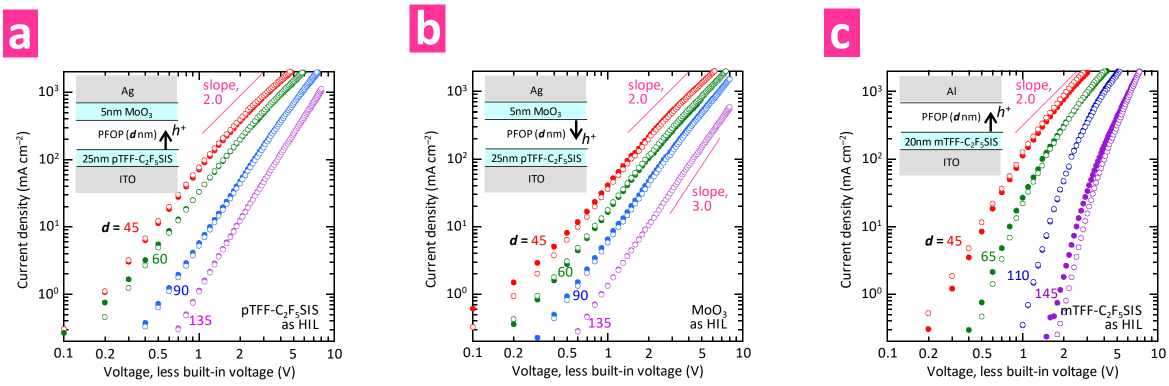

Supplementary Figure 12. Mott–Gurney plots for PFOP semiconductor films with spun-on poly-HIL and evaporated MoO_3 hole contacts. Hole injection from: **(a)** hole-doped pTFF-C_2F_5SIS, or **(b)** MoO_3, in the device structure: glass/ITO/25-nm *p*-doped pTFF-C_2F_5SIS/PFOP/5-nm MoO_3/Ag. **(c)** Hole injection from hole-doped mTFF-C_2F_5SIS in the device structure: glass/ITO/20-nm *p*-doped mTFF-C_2F_5SIS/PFOP/Al. Representative lo-to-hi second sweep data (5 V s^{-1}) for two devices are shown for each. PFOP is poly(9,9-bis(4-octylphenyl)fluorene-2,7-diyl). Thickness is given in plot. MoO_3 was evaporated at 5 x 10^{-6} mbar.

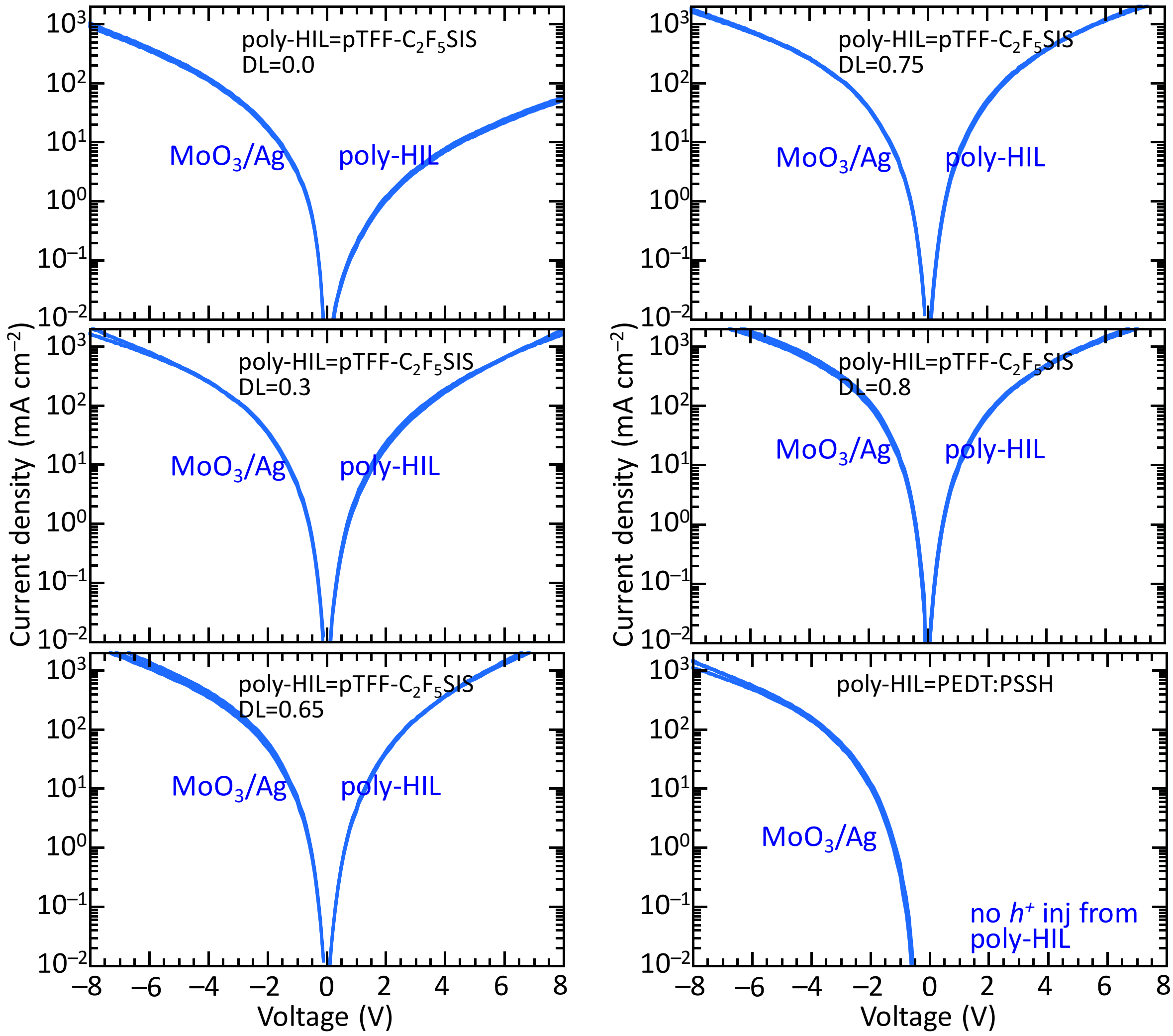

Supplementary Figure 13. Current–voltage characteristics of glass/ITO/25-nm poly-HIL/110-nm PFOP/1.5-nm MoO_3/Ag devices. Poly-HIL is self-compensated, hole-doped pTFF-C_2F_5SIS with selected doping level, as annotated in the plot, or PEDT:PSSH used as reference. The bias voltage on the horizontal axis is applied to the poly-HIL; hole injecting contact is labeled for each branch. Representative lo-to-hi second sweep data (5 V s^{-1}) for two devices are shown for each type. **Doping methodology:** Self-compensated, hole-doped pTFF-C_2F_5SIS solutions were prepared with doping level controlled between 0.0 (undoped) to 0.8 h^+/r.u. by adding the appropriate stoichiometric ratio of oxidant to polymer repeat unit as described in Methods.

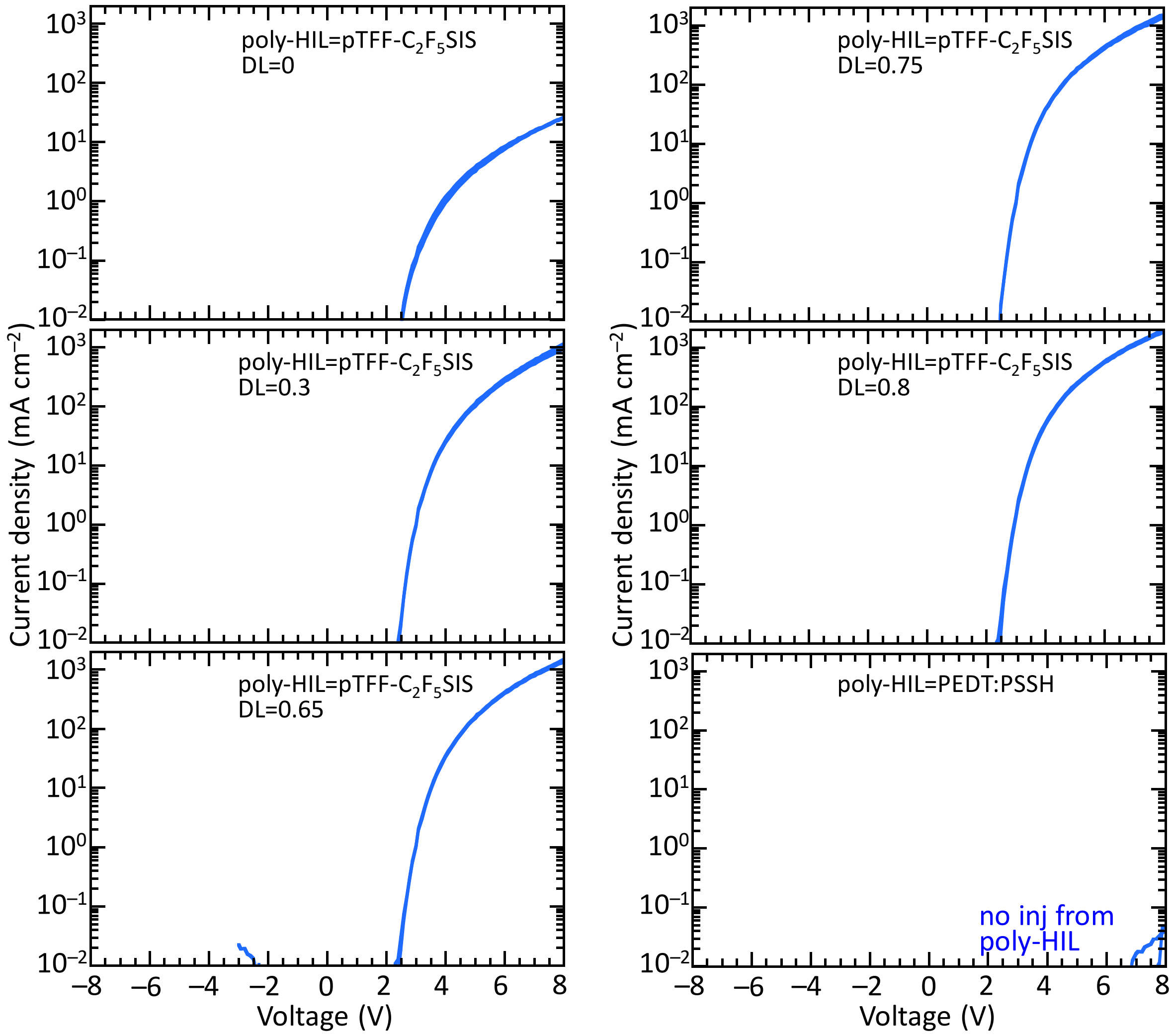

Supplementary Figure 14. Current–voltage characteristics of glass/ITO/25-nm poly-HIL/110-nm PFOP/Al devices. The bias voltage on the horizontal axis is applied to the poly-HIL. Other aspects are same as Supplementary Figure 13.

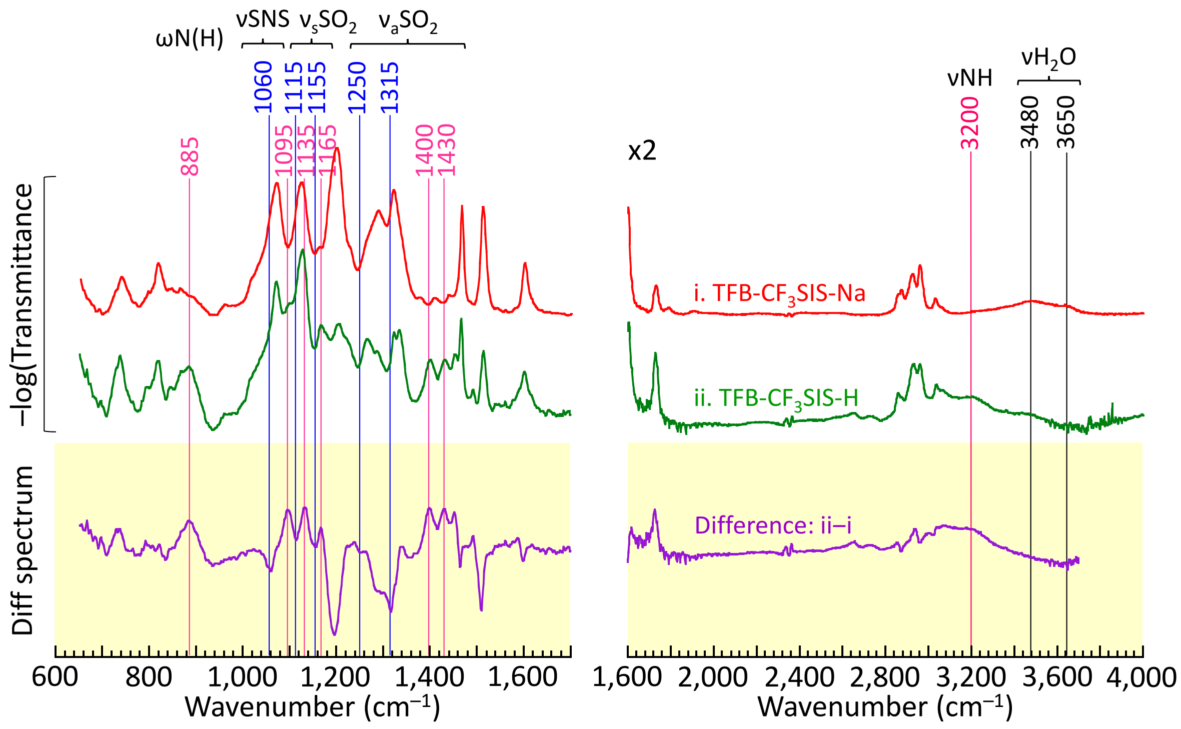

Supplementary Figure 15. Transmission FTIR spectra of TFB-CF_3SIS films. (White panel) Spectra: **(i)** Na^+ salt form, **(ii)** acid (protonated anion) form. **(Yellow panel)** Difference spectra. **Methodology:** TFB-CF_3SIS was synthesized and purified to give the Na^+ salt form, which was spin-cast to give the TFB-CF_3SIS-Na (Na^+ salt) film. This film was contacted with 5-mM trifluoromethanesulfonic acid in methanol for 30 s, and spin-dried to give the TFB-CF_3SIS-H (acid) film. Both films were baked on a hotplate to 220°C for 15 min in nitrogen glovebox before FTIR spectra collection. Blue lines mark the vibrational modes of the imide anion (–$CH_2SO_2N^-SO_2CF_3$); magenta lines, protonated imine anion (–$CH_2SO_2NHSO_2CF_3$); black lines, sorbed molecular water. These assignments have been verified by our DFT/CAM-B3LYP/6-31++G(*d*,*p*) mode frequency analysis, and relate to those of the symmetrical species, $(CF_3SO_2)_2N^-$ and $(CF_3SO_2)_2NH$.[5]

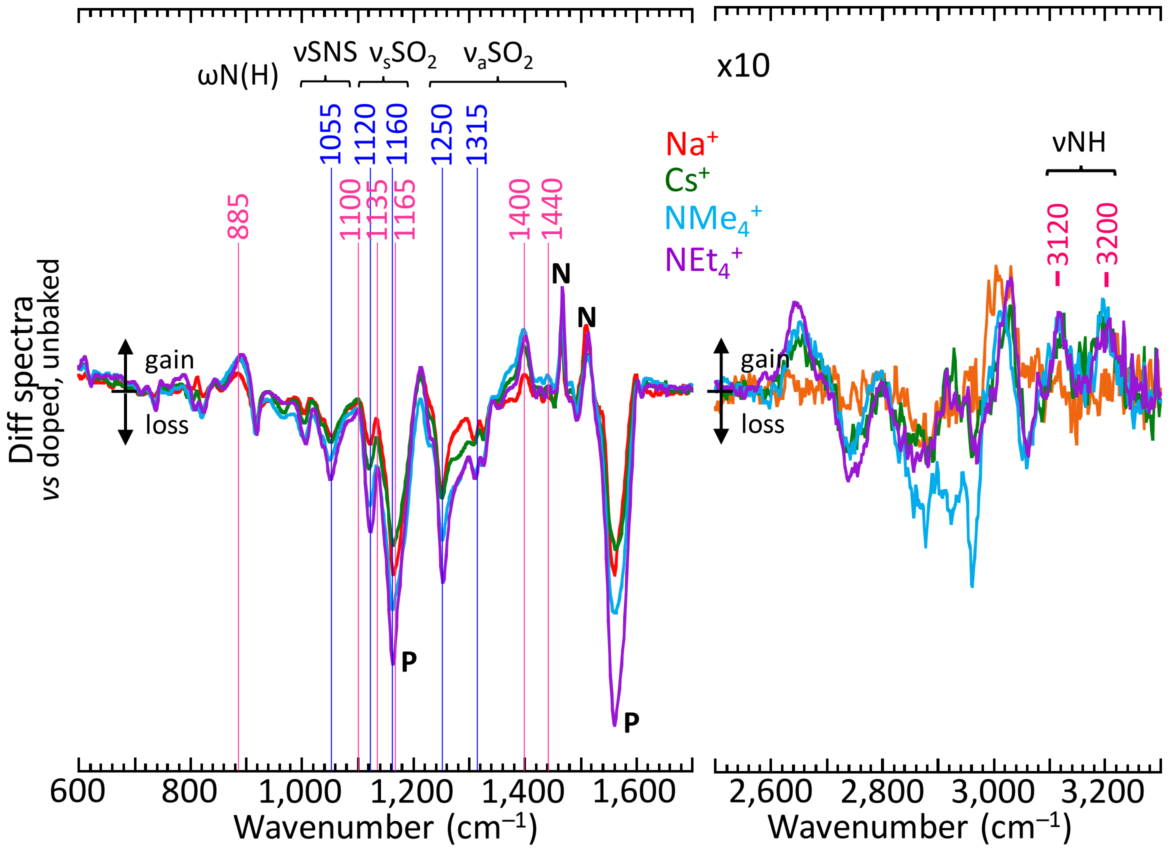

Supplementary Figure 16. Thermal de-doping of hole-doped TFB-CF_3SIS-M films. Transmission FTIR spectra for different spectator cation $M^+ \in \{Na^+, Cs^+, NMe_4^+, \text{and } NEt_4^+\}$, before and after baking on hotplate at 120°C for 10 min in nitrogen glovebox. Difference spectra are referenced to the initial doped film. Blue lines mark the vibrational modes of the imide anion ($–CH_2SO_2N^-SO_2CF_3$); magenta lines, protonated imine anion ($–CH_2SO_2NHSO_2CF_3$). Band labels: **P**, polaron IRAV; **N**, neutral aromatic vibrations. The polaron P_1 absorption beyond 1,800 cm^{-1} has been subtracted for clarity. Initial DL ≈ 0.7 h^+/r.u. Film thickness, 1.5 μm.

Supplementary Table 1-1. Computed gas-phase water dimer $(H_2O)_2$ geometry at DFT/CAM-B3LYP/6-31++G(*d,p*), compared with experiment and higher levels of theory

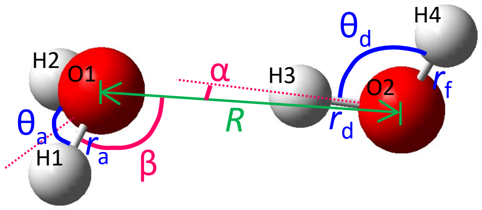

	Method[a]	*R* (Å)	r_d (Å)	r_f (Å)	r_a (Å)	θ_d (°)	θ_a (°)	α (°)	β (°)
1	MP2(FULL)/6-311+G(*d,p*); no-CP; refs[1-3]	2.908	0.965	0.958	0.960	103.6	104.1	2.2	135.8
2	MP2(FULL)/aug-cc-pVTZ; no-CP; ref[4]	2.898	0.965	0.958	0.960	104.4	104.7	0.0	131.1
3	RASSCF & CCSD(T); CP; ref[5]	2.925	0.956	0.951	0.953	105.5	105.2	4.3	128.2
4	MP2(FC)/aug-cc-pVQZ; CP; ref[6]	2.917	0.966	0.958	0.960	104.6	104.7	5.6	124.8
5	CCSD(T)(FULL)/TZ2P(*d,f*)+dif; no-CP; ref[7]	2.909	0.965	0.958	0.960	104.4	104.6	4.7	124.9
6	CCSD(T)/→∞; corrections; best estimate, ref[8]	2.912	0.964	0.957	0.958	104.8	104.9	5.5	124.4
7	Exptal[b]	2.97 +0.00, −0.03						6 ±20	123 ±10
8	This work:[c] CAM-B3LYP/6-31++G(*d,p*)	2.851	0.972	0.962	0.964	106.5	106.5	5.0	128.3

Footnotes:

[a] References: [1] M. J. Frisch, J. E. Del Bene, J. S. Binkley and H. F. Schaefer III, **J. Chem. Phys. 84** (1986) 2279; [2] B. J. Smith, D. J. Swanton, J. A. Pople, H. F. Schaefer III and L. Radom, **J. Chem. Phys. 92** (1990) 1240; [3] J. E. Del Bene, **Int. J. Quantum Chem. Symp. 26** (1992)

527; [4] S. S. Xantheas and T. H. Dunning, Jr, **J. Chem. Phys. 99** (1993) 8774; [5] M. Schütz, S. Brdarski, P.-O. Widmark, R. Lindh and G. Karlström, **J. Chem. Phys. 107** (1997) 4597; [6] P. Hobza, O. Bludský and S. Suhai, **Phys. Chem. Chem. Phys. 1** (1999) 3073; [7] G. S. Tschumper, M. L. Leininger, B. C. Hoffman and H. F. Schaefer III (unpublished); G. S. Tschumper, PhD thesis, University of Georgia, Athens, 1999; [8] W. Klopper, J.G.C.M. van Duijneveldt-van de Rijdt and F.B. van Duijneveldt, **Phys. Chem. Chem. Phys. 2** (2000) 2227

[b] J.A. Odutola and T.R. Dyke, **J. Chem. Phys. 72** (1980) 5062

[c] DFT/ CAM-B3LYP/ 6-31++G(*d,p*) reproduces the intermolecular bond length with error of –0.1 Å *vs* experiment and higher levels of theory, intramolecular bond length and bond angles to better than 0.01 Å and 1.5°, respectively, *vs* higher levels of theory, and intermolecular angles within the uncertainty of experiment and higher levels of theory.

Supplementary Table 1-2. Computed gas-phase water dimer $(H_2O)_2$ dissociation energies at DFT/CAM-B3LYP/6-31++G(*d,p*), compared with experiment and higher levels of theory

	Method	D_e (eV)	D_o (eV)
1	MP2(FULL)/aug-cc-pVTZ; no-CP; ref[1]	0.234	0.156
2	CCSD(T)/→∞; corrections; ref[2]	0.218	0.143
3	CCSD(T)/ aug—cc-pVTZ; hybrid; ref[3]	0.216	0.137
4	Exptal[b]		0.137 ±0.001
5	This work:[c] CAM-B3LYP/6-31++G(*d,p*)	0.291	0.196

Footnotes:

[a] References: [1] S. S. Xantheas and T. H. Dunning, Jr, **J. Chem. Phys. 99** (1993) 8774; [2] W. Klopper, J.G.C.M. van Duijneveldt-van de Rijdt and F.B. van Duijneveldt, **Phys. Chem. Chem. Phys. 2** (2000) 2227; [3] A. Shank, Y.M. Wang, A. Kaledin, B.J. Braams and J.M. Bowman, **J. Chem. Phys. 130** (2009) 144314

[b] B.E. Rocher-Casterline, L.C. Ch'ng, A.K. Mollner and H. Reisler. **J. Chem. Phys. 134** (2011) 211101

[c] DFT/ CAM-B3LYP/ 6-31++G(*d,p*) provides D_e and D_o with error of +75 meV and +60 meV, respectively, *vs* experiment and higher levels of theory. This is well within the expected uncertainty of our methodology (±200 meV).

Supplementary Table 2. Computed vertical and adiabatic ionization energies of gas-phase $(H_2O)_q$ clusters at DFT/CAM-B3LYP/6-31++G(*d,p*), compared with experimental ion appearance energies

q	Computed internal energy for vertical ionization, 0 K (eV)[a]	Computed internal energy for adiabatic ionization, 0 K (eV)[a]	Computed Gibbs free energy for adiabatic ionization, 298 K (eV)[a]	Description of ground (G), and local minimum (I_{loc}) or adiabatic ionized (I_a) states[b]	Exptal ion appearance energy (eV)[c]	Exptal ion product[c]
1				G: monomer	12.62	H_2O^+
	12.73	12.56	12.54	I_a: H_2O^+		
2				G: H-bonded dimer	11.74	H_3O^+
					11.25	$(H_2O)_2^+$
	11.66	10.62	10.64	I_a: $H_3O^+ \ldots {}^\bullet OH$		
3				G: H-bonded cyclic trimer	11.15	$H^+(H_2O)_2$
	11.44	9.82	9.78	I_a: Zundel-like; $(H_2O)_2H^+ \ldots {}^\bullet OH$		
		10.13	10.10	I_{loc}: $(H_2O \ldots OH_2)^{\bullet +}(OH_2)$		
4				G: H-bonded cyclic tetramer	10.94	$H^+(H_2O)_3$
	11.25	9.45	9.35	I_a: Eigen-like; $H_3O^+ \ldots (OH_2)_2({}^\bullet OH)$		
		9.50	9.39	I_{loc}: Eigen-like; $H_3O^+ \ldots (OH_2)(OH_2 \ldots {}^\bullet OH)$		
5				G: H-bonded cyclic pentamer	10.94	
	10.96	9.26	9.24	I_a: Zundel-like; $H_5O_2^+$ in C_2 cyclic pentamer		
		9.29	9.26	I_{loc}: Eigen-like; H_3O^+ cyclic tetramer		
		9.44	9.40	I_{loc}: Zundel-like; cyclic pentamer $(H_2O \ldots OH_2)^{\bullet +} \ldots (OH_2)_2 \ldots OH_2$		

Footnotes:

[a] Computed as the gas-phase energy difference between $M^{\bullet+}$ and M. The vertical energies are computed with both $M^{\bullet+}$ and M in the same optimized ground-state geometry: $(H_2O)_q$ $_{(g)}$ → $(H_2O)_q^+$ $_{(g)}$ + e^- $_{(g)}$. The adiabatic energies are computed with $M^{\bullet+}$ and M in separately optimized geometries, which results in geometric relaxation of the first formed $(H_2O)_q^+$ to a hydronium ion by H-bonding reorganization: $(H_2O)_q$ $_{(g)}$ → $H^+(H_2O)_{q-1}$ $^{\bullet}OH$ $_{(g)}$ + e^- $_{(g)}$. Internal energies are corrected for zero-point energies. Gibbs free energies are computed from the standard expression: $\Delta G = \Delta U + p\Delta V - T\Delta S$, where ΔS is computed at the same DFT level, neglecting thermodynamic quantities pertaining to e^- (g).

[b] $HO^{\bullet}$ is present in the computed final ionized state, but relatively weakly bound and hence easily eliminated in experiments.

[c] Ion appearance energies are from L. Belau, K.R. Wilson, S.R. Leone, M. Ahmed, J. Phys. Chem. A, 111 (2007) 10075, and S. Barth, M.
Ončák, V. Ulrich, M. Mucke, T. Lischke, P. Slavíček, U. Hergenhahn, J. Phys. Chem. A, 113 (2009) 13519. The ion product is given in the last column, based on mass spectrometry measurements. The appearance energy is the threshold photon energy for the appearance of the specified ion product. This gives the maximum limit for adiabatic ionization energy of the neutral cluster. The appearance energy is larger than the photoemission threshold due to production of a “hot” state that subsequently eliminates $HO^{\bullet}$. Comparison of our computed internal energies with the experimental appearance energies show that the latter are typically just below the vertical ionization energies, but well above the adiabatic ionization energies.

Supplementary Table 3. Computed gas-phase adiabatic electron-detachment energies for P^+ $X^-(H_2O)_p$ complexes

S/N	X^-	$\Delta G_{D,g}$ (eV),[a] and product[b]			
		$p = 0$	1	3	5
1	PF_6^-	10.93 A	9.68 M	8.20 WH	8.00 WH
2	$(CF_3SO_2)_2N^-$	8.37 A	8.52 M	8.42 WH	8.32 WH
3	$(CH_3SO_2)(CF_3SO_2)N^-$	7.91 A	7.89 M	8.17 WH	8.14 WH
4	$(C_2F_5SO_2)_2N^-$	8.44 A	8.61 M	8.47 WH	8.35 WH
5	$(CH_3SO_2)(C_2F_5SO_2)N^-$	7.89 A	7.92 M	8.18 WH	8.23 WH
6	$CF_3SO_3^-$	8.51 A	8.76 M	8.64 M	7.92 WH
7	$CH_3SO_3^-$	7.88 A	8.13 M	8.02 WA	7.72 WH
8	$CF_3CO_2^-$	8.13 A	8.29 A	8.44 A	8.34 A
9	$CH_3CO_2^-$	7.00 A	7.18 A	7.33 A	7.59 A
10	$CH_3(CH_3O)PO_2^-$	7.39 A	7.55 A	7.80 A	7.86 A

Footnotes:

[a] Gas-phase adiabatic electron-detachment energy of the hydrated anion in the contact ion pair, computed as energy difference at the DFT/CAM-B3LYP/6-31++G(*d*,*p*) level: $\Delta G_{D,g} = \Delta G_f$ $\{P^+ [X (H_2O)_p]^\bullet{}_{(g)}\} - \Delta G_f$ $\{P^+ X^-(H_2O)_p]_{(g)}$, where P^+ is simulated by tetramethylammonium ion (TMA^+) as size proxy for the positively-charged polymer backbone fragment. The geometries of both $TMA^+ [X (H_2O)_p]^\bullet$ and $TMA^+ X^-(H_2O)_p$ are individually optimized for their charge state. The internal energy ΔU is corrected for zero-point energy. The Gibbs free energy is computed at 298.15 K from: $\Delta G = \Delta U + p\Delta V - T\Delta S$, neglecting thermodynamic quantities pertaining to $e^-{}_{(g)}$.

[b] Ground-state hole-trapped product legend: A, radical; M, admixed state; WH, hydronium ion; WA, covalent acid. For $p = 0$, electron detachment occurs solely from the anion to give

the radical (A). For p = 1, electron detachment can occur from the anion to give the anion radical (A), or the anion admixed with its hydrogen-bonded H_2O to give a mixed radical (M). See Supplementary Figures 3 and 4 for molecular orbitals. For $p \gtrsim 3$, electron detachment can additionally occur from the associated water cluster to give hydronium ion (WH), or covalent acid (WA), together with $HO^{\bullet}$.

Supplementary Table 4

Correction for differential Coulomb stabilization by the local ion cluster $\Delta(\Delta G_{Coul})$

S/N	X^-		ΔG_{Coul} and $\Delta(\Delta G_{Coul})$ (eV)[a,b]			Prod[c]
			r = 0	1	3	
1	PF_6^-	ΔG_{Coul}	−3.56±0.11	−3.77±0.24	−3.76±0.21	WH
		$\Delta(\Delta G_{Coul})$	0.0	−0.2±0.25	−0.2±0.25	
		ΔG_{Coul}	−4.67±0.18	−4.32±0.11	−4.11±0.07	WA
		$\Delta(\Delta G_{Coul})$	0.0	0.35±0.2	0.55±0.2	
		$\Delta(\Delta G_{Coul})$	0.0	(0.35)	(0.55)	A
2	$(CF_3SO_2)_2N^-$ [also $(CH_3SO_2)(CF_3SO_2)N^-$]	ΔG_{Coul}	−3.13±0.24	−3.30±0.20	−3.86±0.49	WH
		$\Delta(\Delta G_{Coul})$	0.0	−0.15±0.3	−0.75±0.55	
		$\Delta(\Delta G_{Coul})$	0.0	(0.25)	(0.4)	A
3	$CH_3SO_3^-$ [also $CF_3SO_3^-$]	ΔG_{Coul}	−3.95±0.19	−4.50±0.23	−5.43±0.24	WH
		$\Delta(\Delta G_{Coul})$	0.0	−0.55±0.3	−1.5±0.3	
		ΔG_{Coul}	−5.21±0.08	−4.84±0.16	−5.72±0.22	WA
		$\Delta(\Delta G_{Coul})$	0.0	0.35±0.2	−0.5±0.25	
		$\Delta(\Delta G_{Coul})$	0.0	(0.35)	(−0.5)	A
4	$CH_3CO_2^-$ [also $CF_3CO_2^-$]	ΔG_{Coul}	−6.07±0.19	−5.31±0.31	−5.10±0.08	WA
		$\Delta(\Delta G_{Coul})$	0.0	+0.75±0.35	+0.95±0.2	
		$\Delta(\Delta G_{Coul})$	0.0	(0.75)	(0.95)	A
5	$CH_3(CH_3O)PO_2^-$	$\Delta(\Delta G_{Coul})$	0.0	(0.4)	(0.5)	A

Footnotes:

[a] $\Delta(\Delta G_{Coul})$ accounts for the Coulomb effects of the spectator ion pairs in the local ion cluster $P^+ X^-(Na^+X^-)_r$ on the free energy of formation of the hole-trapped product, allowing for ion cluster reorganization, where P^+ is simulated by the tetracyclic tetraalkylene ammonium model ion $NC_{20}H_{36}^+$ to impose an effective van der Waals radius of 3.5 Å.

[b] **Computation methodology.** PM3 was employed for realistic ion–ion geometry optimization and Coulomb energy calculations. For hydronium calculations (i.e. WH product), we used H_3O^+ as model. **Setup:** In a typical calculation, a series of configurations of P^+ $X^-(Na^+X^-)_r$, where P^+ = $NC_{20}H_{36}^+$, were generated by MM2 molecular dynamic simulations at 150 K, background dielectric constant 1.5, simulation step size 1 fs, with snapshot interval 10 ps. Each configuration was then optimized by PM3 at 0 K to give a set of local-minimum configurations $\{[P^+ X^-(Na^+X^-)_r]_{loc1,i}\}$. **WH and WA products:** For each configuration $[P^+ X^-(Na^+X^-)_r]_{loc1,i}$, we computed ΔH_f, then randomly placed one H_3O^+ at the cluster surface in the vicinity of an anion, re-optimized cluster geometry, and computed ΔH_f for the corresponding hydronium-attached structure $[P^+ X^-(Na^+X^-)_r\ H_3O^+]_{loc,i}$. Then we removed the H_3O^+, re-optimized cluster geometry, and re-computed ΔH_f for the neutral ion cluster structure, denoted $[P^+ X^-(Na^+X^-)_r]_{loc2,i}$. This second neutral configuration is related to the first by addition and removal of H_3O^+. Together they form an adiabatic pair of configurations related to the attachment of H_3O^+. We computed the average change in enthalpy ΔH at 0 K for binding of H_3O^+ to the ion cluster in the local adiabatic approximation: $\Delta H = \Delta H_f\ [P^+ X^-(Na^+X^-)_r H_3O^+]_{loc,i} - ½ * \{\Delta H_f\ [P^+ X^-(Na^+X^-)_r]_{loc1,i} + \Delta H_f\ [P^+ X^-(Na^+X^-)_r]_{loc2,i}\} - \Delta H_f\ [H_3O^+]$. This algorithm was repeated for different H_3O^+ placements, and cluster configurations i, to yield a ΔH distribution. For WH product, ΔH is typically negative, indicating stabilization of the hydronium by the ion cluster due to more favorable Coulomb interactions in the final state. For WA product, however, ΔH is often positive, indicating destabilization of the covalent acid formation by the ion cluster due to less favorable Coulomb interactions in the final state. Then we 'cleaned' the data to eliminate artefacts. We eliminated all hydronium-attached configurations with ΔH_f more than 0.6 eV positive of the most negative value. These are local structures trapped in high-energy minima. We also eliminated outliers more than three standard deviations from the mean. For some anions, $CH_3SO_3^-$ and $CH_3CO_2^-$, the calculations yield a covalent-acid species X–H, which we classified and processed separately. **A products:** For each $[P^+ X^-(Na^+X^-)_r]_{loc1,i}$ configuration, we removed an electron, re-optimized cluster geometry using unrestricted Hartree–Fock PM3 to get the electron-detached structure $[P^+ X^-(Na^+X^-)_r - e^-]_{loc,i}$. We then re-added the e^-, re-optimized cluster geometry, and re-computed ΔH_f for the neutral ion cluster structure, denoted $[P^+ X^-(Na^+X^-)_r]_{loc2,i}$. We computed the average change in enthalpy ΔH at 0 K for binding of e^- to the ion cluster in the local adiabatic approximation, as before: ΔH =

ΔH_f [P^+ $X^-(Na^+X^-)_r$ – e^-]$_{loc,i}$ – ½ * {ΔH_f [P^+ $X^-(Na^+X^-)_r$]$_{loc1,i}$ + ΔH_f [P^+ $X^-(Na^+X^-)_r$]$_{loc2,i}$]}. Such calculations often encounter convergence problems, in which case, we replaced the final electron-detached configuration with the neutral X–H, which has same charge and similar steric size, i.e. WA results. For A product, ΔH is also often positive, indicating destabilization of radical formation by the ion cluster due to less favorable Coulomb interactions in the final state. **Estimation of $\Delta(\Delta G_{Coul})$.** We then take the difference in ΔH to estimate the effect of the spectator ion pairs on the formation of the hole-trapped product: $\Delta(\Delta H)(r)$ = $\Delta H(r)$ – $\Delta H(r{=}0)$. Since this quantity is dominated by ion–ion Coulomb interactions, we denote it $\Delta(\Delta H_{Coul})$. Neglecting the (small) entropy term, this corresponds also to $\Delta(\Delta G_{Coul})$ given by $\Delta G_{Coul,f}$ – $\Delta G_{Coul,i}$. Values in brackets are assumed.

[c] Ground-state hole-trapped product legend:

WH = hydronium ion and HO•, from water oxidation reaction

WA = covalent acid (i.e. protonated anion) X–H and HO•, from water oxidation reaction

A = X•, from anion oxidation reaction

Supplementary Note 1. Chemical density-of-states (DOS)

Consider a putative chemical trap, one of the species identified in Eqs (2) to (5) in the main article, hosted by a local ion cluster. The composition and configuration of the trap vary with time, due to reorganization in ion-containing soft materials. The composition is given by the number of water molecules p associated with the anion, and number of spectator ion pairs r in the local ion cluster. The configuration is given by the arrangement of these water molecules and ions. The relevant energy for hole trapping is the solid-state adiabatic electron-detachment Gibbs free energy $\Delta G_{D,s}$ of the trap, marked on the vacuum energy scale as $-E_t$ in the standard semiconductor picture. Negative direction points downwards, following standard electron convention. Thus, E_t gives the minimum energy required to remove an electron from the hole trap to the vacuum level.

However, E_t depends on both the composition and configuration of the trap. For fixed composition, the configuration can vary dynamically with time. The relevant observation time scale τ is about 10 h for processing stability, but longer for shelf stability. We denote the Gibbs free energy of formation of the trap configuration as E, which provides a convenient label. In particular, multiple initial configurations are 'frozen in' with different E values at the point of film formation, and each of these continually anneal towards lower E during τ. But by accessing different degrees of hydration, the trap configuration may also jump to a higher E.

Let us define the notion of chemical DOS, $D(E_t)$, as the distribution of E_t of the chemical trap energy per unit energy interval per unit volume of material, which evolves over time t. For each (p, r) composition, there is a distribution of $E(p,r)$ due to configuration variations that are also time dependent. For each $E(p,r)$, there is a corresponding E_t. Thus, the normalized integral gives the single-trap chemical DOS, i.e. $\int_{-\infty}^{\infty} N(E_t, E(p,r))\, dE(p,r) = 1$, at time t. The weighted sum over all (p, r) attained at the given temperature and humidity gives the chemical DOS, i.e. $D(E_t) = \sum_{p,r} g_{p,r} N(E_t, E(p,r))$, where $g_{p,r}$ is the number density of traps with composition (p, r) at t. Through Ostwald ripening, individual trap compositions may increase in r with t.

We do not need to compute $D(E_t)$. For evaluation of stability, we only need to compute the frontier edge of this distribution accessible within the observation time. In the long-time limit, the trap configurations can be estimated by gas-phase energy minimization after suitable corrections for the solid state.

The electron transfer rate, which is of the order of nanoseconds, is fast compared to the reorganization rate of the water and ion clusters, which is of the order of microseconds for water diffusion, and much longer for ion rearrangements. Thus, formation of the trap configuration is rate limiting. As soon as one forms with E smaller than ϕ, chemical hole trapping occurs.

Finally, a comment about the hole DOS at the Fermi level, $D_h(E_F)$. This is given by $\frac{dn_h}{dE_F}$, which is the change in hole density with change in Fermi energy. The n_h *vs* E_F characteristics have been measured for the triarylaminium family of polymers.[6] In the heavily-doped regime, ϕ rises 0.1 eV per decade of hole doping. Saturation doping occurs at $n_h \approx 8 \times 10^{20}$ cm^{-3}. Hence $D_h(E_F)$ is *ca*. 8×10^{21} cm^{-3} eV^{-1}. Because of strong electron correlation, this is not equal to the spectroscopically measured DOS at the EL itself, which in fact is close to zero.[6]

Supplementary Note 2. Matrix polarization and carrier screening

Matrix polarization was computed by volume integration of the electrostatic field in a polarizable continuum model:[7-9] $U_{\mathrm{el}} = \int_{\mathrm{vol}} \frac{e^2}{32\pi^2\varepsilon_o\varepsilon_r}\frac{1}{r^4}\,dV$, where dV is the volume element, and r is its distance from the effective positive point-charge created in the cluster, integrating over the volume outside of the local ion cluster and the P^+ model. The dielectric constant of the matrix ε_r is taken to be 3.0. The polarization energy due to the medium $\Delta G_{\mathrm{pol,m}}$ is then computed as the difference between this quantity and the corresponding one in vacuum where ε_r is 1. For WA and A pathways, we take the positive charge in the final state to reside on the anion-under-evaluation. For WH pathway, we treat the hydronium at surface of the ion cluster, at the interface with the hydrophobic matrix. We adopt the following effective Born radii:[10,11] $H_5O_2^+$ $HO^\bullet$, 2.65 Å; and $H_3O^+(H_2O)_3$ $HO^\bullet$, 3.0 Å, which we deduced from their literature solvation energies in water. For hydronium ions embedded fully inside the hydrophobic matrix, we get $\Delta G_{\mathrm{pol,m}}$ of −1.6 and −1.8 eV, for p = 3 and 5, respectively.

The polarization energy due to carrier screening $\Delta G_{\mathrm{pol,sc}}$ is computed also at the same level of approximation for a screening radius R_s beyond which ε_r is taken to be infinity: $\Delta G_{\mathrm{pol,sc}} = -\frac{1}{2}\cdot\frac{e^2}{4\pi\varepsilon_o}\left(\frac{1}{\varepsilon_r}\right)\frac{1}{R_S}$. For a hole density $n_h \approx 0.6 \times 10^{21}$ cm^{-3} (corresponding to DL ≈ 0.8 h^+/r.u.), R_S is estimated by $n_h^{-1/3}$ to be 12 Å, which gives $\Delta G_{\mathrm{pol,sc}}$ = −0.2 eV. All hole-trapping pathways require this correction because of the $|1e|$ positive charge in the final but not initial state. The polarization stabilization of the initial state is approximated to zero. Hence, $\Delta(\Delta G_{\mathrm{pol}}) \approx \Delta G_{\mathrm{pol,f}} = \Delta G_{\mathrm{pol,m}} + \Delta G_{\mathrm{pol,sc}}$.

Supplementary References

1 Gaiduk, A. P., Pham, T. A., Govoni, M., Paesani, F. & Galli, G. Electron affinity of liquid water. *Nat. Commun.* **9**, 247 (2018).

2 Coe, J. V., Williams, S. M. & Bowen, K. H. Photoelectron spectra of hydrated electron clusters vs cluster size: connecting to bulk. *Int. Rev. Phys. Chem.* **27**, 27-51 (2008).

3 Herbert, J. M. & Coons, M. P. The hydrated electron. *Annu. Rev. Phys. Chem.* **68**, 447-472 (2017).

4 Png, R. Q. *et al.* Electromigration of the conducting polymer in organic semiconductor devices and its stabilization by crosslinking. *Appl. Phys. Lett.* **91**, 013511 (2007).

5 Yu, L., Pizio, B. S. & Vaden, T. D. Conductivity and spectroscopic investigation of bis(trifluoromethanesulfonyl)imide solution in ionic liquid 1-butyl-3-methylimidazolium bis(trifluoromethanesulfonyl)imide. *J. Phys. Chem. B* **116**, 6553-6560 (2012).

6 Png, R. Q. *et al.* Madelung and Hubbard interactions in polaron band model of doped organic semiconductors. *Nat. Commun.* **7**, 11948 (2016).

7 Rips, I. & Jortner, J. Ion solvation in clusters. *J. Chem. Phys.* **97**, 536-546 (1992).

8 Zhan, C. G., Zheng, F. & Dixon, D. A. Theoretical studies of photoelectron spectra of $SO_4^{2-}(H_2O)_n$ clusters and the extrapolation to bulk solution. *J. Chem. Phys.* **119**, 781-793 (2003).

9 Winter, B. *et al.* Electron binding energies of aqueous alkali and halide ions: EUV photoelectron spectroscopy of liquid solutions and combined ab initio and molecular dynamics calculations. *J. Am. Chem. Soc.* **127**, 7203-7214 (2005).

10 Still, W. C., Tempczyk, A., Hawley, R. C. & Hendrickson, T. Semianalytical treatment of solvation for molecular mechanics and dynamics. *J. Am. Chem. Soc.* **112**, 6127-6129 (1990).

11 Im, W., Lee, M. S. & Brooks III, C. L. Generalized Born model with a simple smoothing function. *J. Comput. Chem.* **14**, 1691-1702 (2003).
